# The effects of NCBP3 on METTL3‐mediated m6A RNA methylation to enhance translation process in hypoxic cardiomyocytes

**DOI:** 10.1111/jcmm.16852

**Published:** 2021-08-12

**Authors:** Fei Ye, Xiaoyan Wang, San Tu, Lixiong Zeng, Xu Deng, Wenzhi Luo, Zhihui Zhang

**Affiliations:** ^1^ Department of Cardiology The Third Xiangya Hospital of Central South University Changsha China

**Keywords:** cardiomyocytes, eIF4A2, hypoxia, m6A RNA methylation, METTL3, NCBP3, translation

## Abstract

Hypoxia as a crucial pathogenesis factor usually results in huge harmful effects on cardiac injury and dysfunction. Our previous study has uncovered the global transcriptome and translatome profiles of cardiomyocytes in vitro and in vivo to response to hypoxia by RNA sequencing and ribosome profiling sequencing. We observe a series of differential expressed genes between transcription and translation, which may be attributed to the hypoxia‐specific binding affinity of nuclear cap‐binding subunit 3 (NCBP3) at 5' untranslation region of target genes. Although we observe that NCBP3 can facilitate translational process in myocardium under hypoxia stress, the underlying molecular mechanism of NCBP3 for gene translation modulation remains unclear. In this study, we performed NCBP3 immunoprecipitation for mass spectrum and found that METTL3 and eIF4A2 particularly interacted with NCBP3 in hypoxic rat H9C2 cardiomyocytes. Furthermore, we observed that METTL3‐mediated N6‐methyladenosine (m6A) methylation was elevated in hypoxia, but compromised by NCBP3 or METTL3 knockdown. Finally, we also demonstrated that NCBP3/METTL3/eIF4A2 regulatory axis plays a specific role in cardiomyocytes undergoing hypoxic stress. Taken together, we unmasked NCBP3, a novel hypoxia‐specific response protein functions as a scaffold to coordinate METTL3 and eIF4A2 for enhancing gene translation by m6A RNA methylation in cardiomyocytes upon hypoxic stress.

## 1 INTRODUCTION

According to the World Health Organization (WHO), cardiovascular disease (CVD) causes 1.75 million deaths accounting for 30% global deaths every year.^1^ Acute myocardial infarction (AMI) has become the leading cause of CVD. Myocardial ischaemia and hypoxia result in cardiomyocyte apoptosis and necrosis and ultimately lead to impaired cardiac function. In turn, recent scholars have indicated that hypoxia can reduce ROS generation and benefit cardiomyocyte proliferation, even induce heart regeneration in myocardium infarction models.^2^, ^3^ The debate over whether hypoxia benefits or harms cardiomyocyte proliferation has never been resolved, and the underlying mechanism still remains obscure.

Cells experience a series of physiological changes to adapt hypoxic stress via shifting cellular processes from general housekeeping functions to specialized hypoxia–response pathways activation. Transcriptomic changes responding upon hypoxia are relatively well illustrated, whereas gene translation as one important regulatory means still remains a mystery. There are several proposed hypoxic translation machineries: open reading frame‐mediated mRNA regulation^4^ endoplasmic reticulum‐mediated mRNA selection^5^ IRES‐dependent translation initiation^6^, ribosomal hypoxia–response elements (rHRE) in the mRNA^7^ and RNA binding proteins recruitment at UTRs for mRNA stabilization.^8^

To monitor the localized protein synthesis and explore cotranslational folding and targeting, ribosome profiling technique (polyribo‐seq) based on sucrose‐gradient separation of polysome‐associated RNAs is allowed to assess the coding potential of mRNAs.^9^ Our previous study^10^ has compared the global transcriptome and translatome in hypoxic myocardium in vitro and in vivo via RNA‐seq and polyribo‐seq. A large number of differential expressed genes between transcriptome and translatome indicate the complicated post‐transcriptional and translational reactions responding to hypoxia. We have found that nuclear cap‐binding subunit 3 (NCBP3) specifically occupies at 5' UTR of genes which are up‐regulated in translatome compared with transcriptome. NCBP1, 2 and 3 constitute the cap‐binding complex that binds all capped RNA and are necessary for RNA processing and intracellular localization.^11^ NCBP3 has been demonstrated to engage in interacting with components of the exon junction complex and transcription and export complex including eIF4A3, MAGOH, RBM8A ALYREF and DDX39B to enhance mRNA biogenesis.^12^ However, the current understandings of NCBP3 for gene translation in hypoxic condition are unknown. In this study, we continue with the investigation of myocardial ischaemia and focus on the function of NCBP3 on gene translational process, as well as the connection between NCBP3 and hypoxia. The data may help us to deeply understand the regulatory network of gene translation responding to hypoxia.

## 2 MATERIALS AND METHODS

### 2.1 Cell culture

Rat H9C2 cardiomyocytes (Cell Bank of Shanghai Institutes of Biological Sciences, Shanghai, China), human 293T embryonic kidney cells, glioma U251 cells, prostate cancer LNCapPC3 cells, non‐small‐cell lung cancer A549 cells and AC16 cardiomyocytes (Type Culture Collection of the Chinese Academy of Sciences, Beijing, China) were cultured in Dulbecco's modified Eagle's medium (Thermo Fisher Scientific) with 10% foetal bovine serum (Thermo Fisher Scientific). Nucleotides of NCBP3 siRNA (5'‐UGUUCUUUCUUUUCAAUUGCU‐3', 5'‐CAAUUGAAAAGAAAGAACAGC‐3') and METTL3 siRNA (5'‐AAAUUUGCCCAAGAUACUGAC‐3', 5'‐CAGUAUCUUGGGCAAAUUUGC‐3') were synthetized by GenePharma. SiRNAs or plasmids were transfected into these cells using Neon transfection system (Thermo Fisher Scientific) according to the manufacturer's instructions. Cells were cultivated in hypoxia workstation (INVIVO2 400, Ruskinn) with the conditions of hypoxia (1% oxygen) or normoxia (21% oxygen) for 24 h and reoxygenation. Cells were harvested after 6 h reoxygenation for the consequent experiments.

### 2.2 Immunoprecipitation (IP)

The whole‐cell lysates or nuclear extracts were mixed with 1 μg NCBP3 (ab91556, Abcam), METTL3 (ab195352, Abcam), eIF4A2 (ab31218, Abcam) or IgG rabbit IgG antibody, and 40 μl flurry IgA beads (Thermo Fisher Scientific) for rotating overnight at 4°C. Immunoprecipitates were washed by IP buffer (20 mmol/L HEPES [pH 7.9], 350 mmol/L NaCl, 0.1% NP‐40, 1 mmol/L DTT, 0.2 mmol/L PMSF, 2 mg/ml leupeptin and 2 mg/ml aprotinin) and purified by RIPA buffer (50 mmol/L Tris [pH 7.4], 150 mmol/L NaCl, 1% NP‐40, 0.5% sodium deoxycholate, 0.1% SDS) with 1% proteasome inhibitor cocktail and 1% PMSF. The protein lysate was processed by Western blot assay.

### 2.3 Gel filtration assay

Gel filtration assay was performed as previously described.^13^ In brief, hypotonic buffer with NP‐40 was used to separate the cytoplasm and nuclei of 1x10^8^ GT1‐7 cells,the nuclear proteins were isolated by high salt extraction buffer [20 mmol/L HEPES (pH 7.9), 420 mmol/L Nacl, 25% glycerol, 1.5 mmol/L MgCl_2_, 0.2 mmol/L EDTA, 0.5 mmol/L dithiothreitol and protease inhibitors]. The nuclear extracts (4 mg) were directly applied to a sepharose 6B column (Sigma‐Aldrich) equilibrated with column running buffer containing 20 mmol/L HEPES (pH 7.9), 200 mmol/L NaCl, 1 mmol/L dithiothreitol, 0.1 mmol/L phenylmethylsulfonyl fluoride and 10% glycerol. Each fractions with 1 ml were collected and detected NCBP3, METTL3 and eIF4A2 by Western blot assay.

### 2.4 Western blot (WB) assay

The protein lysate was subjected to SDS/PAGE and transferred to PVDF membranes (Bio‐Rad Laboratories). The membrane was blocked with 5% fat‐free milk in PBST for 30 min, followed by incubation overnight at 4°C with final dilutions of primary antibodies against NCBP3, METTL3, eIF4A2 or GAPDH (#60004‐1, Proteintech Group). After that, the membrane was washed three times and then incubated with HRP‐conjugated secondary antibodies (Proteintech Group). The blotting bands were developed with ECL plus immunoblotting detection reagents (Thermo Fisher Scientific) and captured using ImageJ.

### 2.5 Liquid chromatography–tandem mass spectrometry (LC‐MS/MS)

Enzymatic hydrolysation was initially conducted by 12 ng/µl trypsin at 37°C for 16 h. Whole peptides were dissolved within 0.1% formic acid and passed through RP‐C18 column for desalination and washed by 0.1% formic acid–acetonitrile. Peptide library was processed through trap column, analysis column and RP‐C18 column by EASY‐nLC1200 system. Enzymatic peptides were analysed using Q‐Exactive^TM^. 300–1600 m/z precursor scan was performed after standard solution adjustment. Full scan model of data‐dependent acquisition was conducted followed by MS2 scan; for MS1 phase, resolution: 35,000, AGC target: 1 x 10^6^, max injection time: 50 ms; for MS2 phase, resolution: 17,500, AGC target: 2 x 10^4^, max injection time: 30 ms. The peptides were aligned by ProteomeDiscover 2.3 (MS1 tolerance 10 ppm, MS2 tolerance 0.02 Da, missed cleavage 2, static modification carbamidomethyl, dynamic modification acetyl (protein N‐term), deamidated (NQ), oxidation (M)). Differential expressed genes were evaluated (fold change >2 or <0.5, *p* < 0.05).

### 2.6 Dot plot

Dot plot was performed as previously described.^14^ In brief, total RNA or poly (A) + mRNA was isolated as described above. RNA samples dissolved in 3 times volume of RNA incubation buffer were denatured at 65°C within 5 min. Then, the samples, divided into subgroups of 400, 200 and 100 ng, were loaded to an Amersham Hybond‐N+ membrane (GE Healthcare) installed in a Bio‐Dot Apparatus (Bio‐Rad Laboratories) with the mixture of ice‐cold 20*SSC buffer (Millipore). The membrane was UV crosslinked for 5 min and washed with PBST. Whereafter, it was stained with 0.02% methylene blue (Shanghai Sangon Biotechnology Company), followed by the scanning to indicate the total content of input RNA. After being blocked with 5% non‐fat milk, the membrane was incubated with specific m6A antibody (1:1000, Millipore) overnight at 4°C. Dot blots were hatched with HRP‐conjugated anti‐mouse immunoglobulin G (IgG) for 1 h before visualized by an imaging system (Bio‐Rad Laboratories).

### 2.7 RNA immunoprecipitation (RIP) assay

RNA immunoprecipitation or MeRIP experiments were conducted as previously described.^15^ In brief, 1 × 10^7^ H9C2 cells were harvested, resuspended in nuclear isolation buffer (1.28 mol/L sucrose, 40 mmol/L Tris pH 7.5, 20 mmol/L MgCl2, 4% Triton X‐100) and kept on ice for at least 30 min with frequent mixing. The pellet nuclei were centrifuged with 16000 *g* for 15 min, resuspended by wash buffer (150 mmol/L KCl, 25 mmol/L Tris pH 7.4, 5 mmol/L EDTA, 0.5 mmol/L DTT, 0.5% NP‐40, 100 U/ml RNAase inhibitor (Solarbio), 1× Protease inhibitors cocktail (Solarbio) and sheared the chromatin through sonication by high power, 5 s on, 30 s off for 30 cycles. After that, 90% nuclei were incubated with 1 μg m6A, NCBP3, METTL3 or eIF4A2 antibodies overnight and 40 μl protein A/G beads (Thermo Fisher Scientific) 2 h by gentle rotation at 4°C while the rest of 10% were harvested as input. The pellet beads were centrifuged by 1200 *g* 3 min, washed three times. Both the input and pellet beads were purified by RNAiso plus (Takara) and conducted reverse transcription using QuantiTect Reverse Transcription Kit (Qiagen).

For high‐throughput sequencing, the concentration and quality of purified RIP RNAs were measured by Nanodrop 2000 (Thermo Fisher Scientific) and Agilent bioanalyser 2100 (Agilent). 4 μg RNA in each group was used for library preparation by NEBNext Ultra Directional RNA Library Prep Kit for Illumina (NEB) following manufacturer's instructions and was sequenced on an Illumina Hiseq platform. The raw data deposited into ArrayExpress database (www.ebi.ac.uk/arrayexpress) with the accession number E‐MTAB‐10682 were trimmed adaptors and filter out low‐quality reads using Trimmomatic (non‐default parameters: SLIDINGWINDOW:4:15 LEADING:10 TRAILING:10 MINLEN:35)^16^ and checked the quality of clean reads using Fastqc.^17^ Next, clean reads were aligned to the latest mouse genome assembly mm10 using Hisat2 v2.0.5 (non‐default parameters: ‐‐rna‐strandness RF ‐‐dta).^18^ The transcripts were assembled and the expression levels were estimated with FPKM values using the StringTie algorithm (non‐default parameters: ‐‐rf).^19^ Differential mRNA and lncRNA expression among the groups was evaluated using an R package Ballgown,^20^ and the significance of differences by the Benjamini & Hochberg (BH) *p*‐value adjustment method was computed. Gene annotation was described by Ensembl genome browser database (http://www.ensembl.org/index.html). The R package ClusterProfiler was used to annotate the differential genes with gene ontology (GO) terms and Kyoto Encyclopedia of Genes and Genomes (KEGG) pathways.^21^ The peaks of m6A at certain gene were browsed by Integrative Genomics Viewer (IGV).

### 2.8 Quantitative polymerase chain reaction assay

Quantitative polymerase chain reaction (qPCR) was performed using CFX Fast real‐time PCR system (Bio‐Rad Laboratories). The following cycle parameters were used for all experiments: 30 s at 94°C for pre‐denaturation, 20 s at 94°C, 30 s at 60°C, and 30 s at 72°C for total 45 cycles. The relative levels of mRNA for each specific gene were normalized to GAPDH. Table S1 shows the sequences for all primer sets used in these experiments.

### 2.9 Statistical analysis

The results were presented as the mean ± SD. The significance of difference among the groups was assessed by Student's *t* test. All analysis was processed by SPSS 20 software. The *p*‐value less than 0.05 was considered as statistically significant.

## 3 RESULTS

### 3.1 Characterization of RNA‐binding proteins interacted with NCBP3 in hypoxic H9C2 cells

**FIGURE 1:**
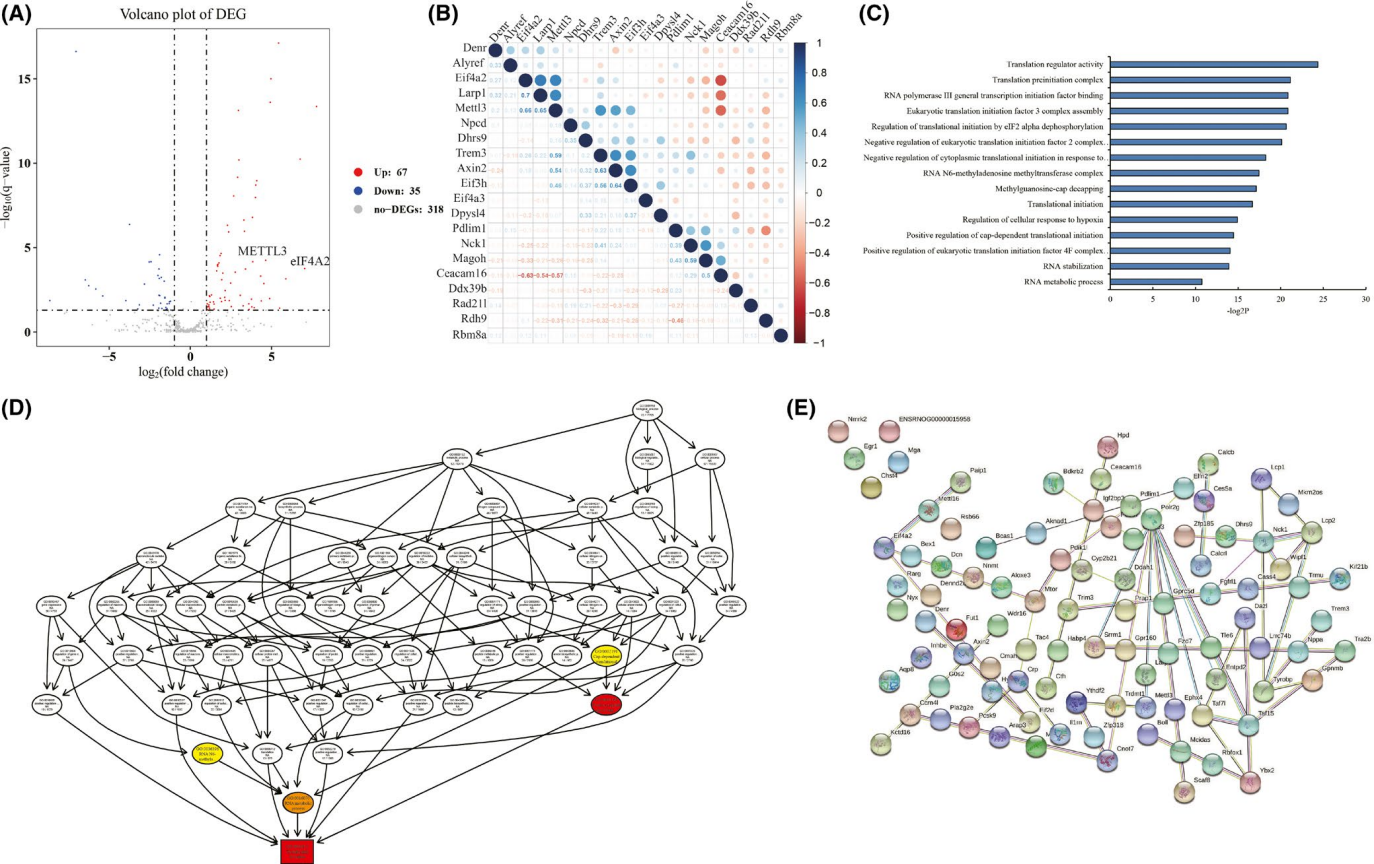
Differentially interacting proteins of NCBP3 between hypoxia and normoxia. (A) Differentially interacting proteins of NCBP3 browsed by volcano plot. Sixty‐seven proteins were higher while 35 were lower in hypoxia compared to normoxia. (B) The correlation with each protein analysed by Pearson's correlation analysis. (C) The enriched functions on differentially interacting proteins by Gene Ontology analysis. (D) The regulatory connection among different functions by directed acyclic graph. (E) Protein–protein interactions among differentially interacting proteins pulled down by NCBP3

Our previous study has unmasked that NCBP3 can specially recognize ‘GAAGCUGCC’ at 5' UTR of mRNAs, and further affects a large number of genes’ translation in hypoxic H9C2 cells.^10^ To further understand the role of NCBP3 in translational process, IP was conducted to pull down NCBP3 (Figure S1A) followed by mass spectrum (Figure S1B). The differential binding protein profiling of NCBP3 between normoxic and hypoxic rat H9C2 cardiomyocytes was identified (log2FC>1 or <−1, *p* < 0.05) (Figure 1A). We found that the binding abilities with NCBP3 strengthened in 67 proteins while weakened in 35 proteins in hypoxic condition compared with normal control (Table S2). The correlations among top 20 differential expressed genes (DEGs) indicated that these binding proteins were likely to function as several complexes to coordinate with NCBP3 in post‐transcriptional regulation responding to hypoxia (Figure 1B). GO analysis suggested that the DEGs were mainly related to RNA metabolism, stabilization, transcription factor activity, RNA polymerase regulation upon hypoxia, translation initiation process, 5' NAD‐cap decapping as well as RNA N6‐methyladenosine (m6A) methyltransferase complex, which was in accord with the probable biological events in our system (Figure 1C). It was noteworthy that m6A RNA methylation and cap‐dependent translational process were shown in the central position within the directed acyclic graph of functional regulation (Figure 1D). Consistently, protein–protein interaction analysis displayed that METTL3 and eIF4A2 located in the central position of protein network and connected with multiple proteins (Figure 1E). Taken together, we preliminary characterized a unique binding protein profiling of NCBP3 in hypoxic H9C2 cells.

### 3.2 Coordination between METTL3 and eIF4A2 by NCBP3 in hypoxic condition

**FIGURE 2:**
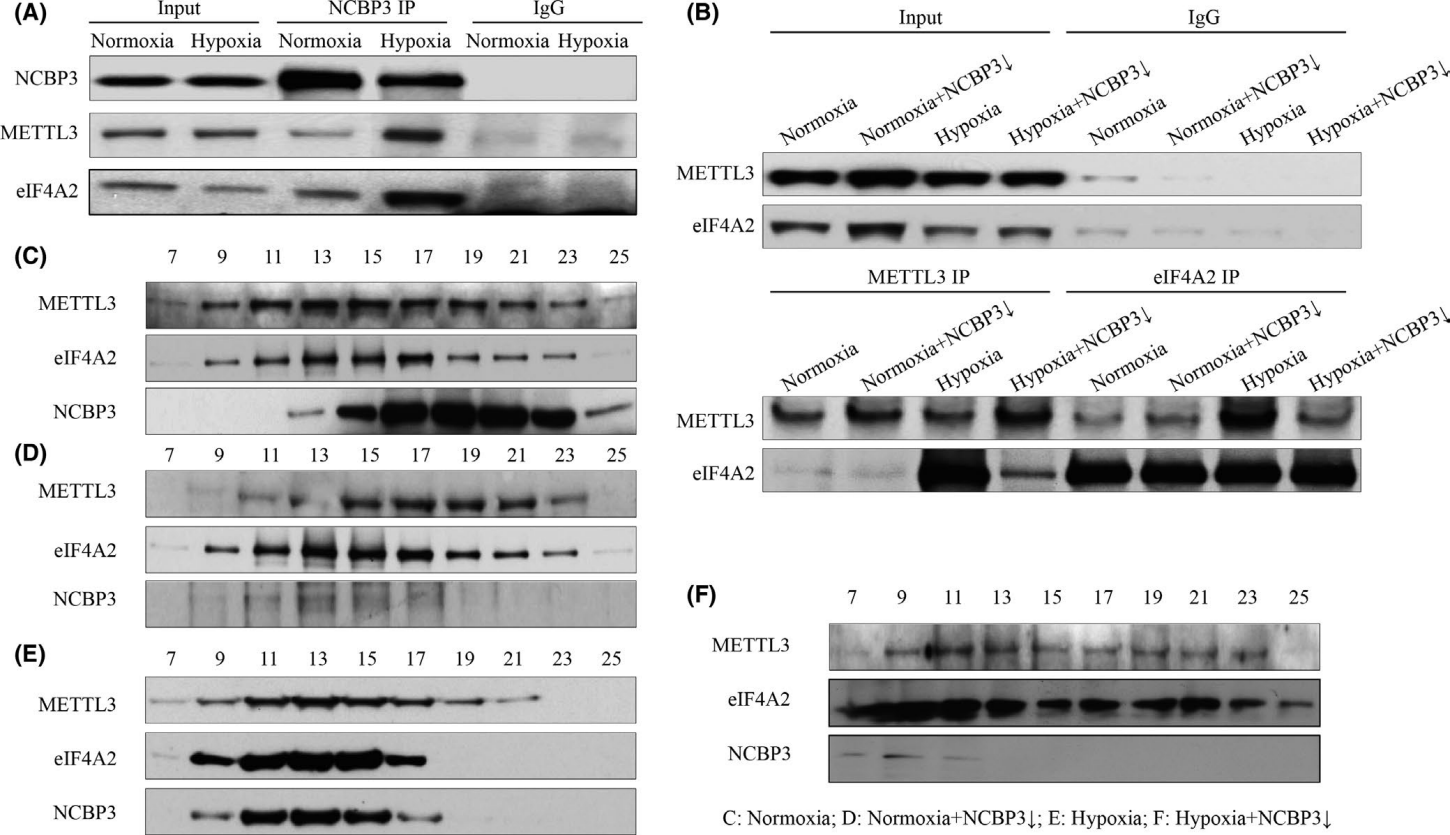
Coordination among NCBP3, METTL3 and eIF4A2 in hypoxic condition. (A) Interaction of NCBP3 with METTL3 and eIF4A2 in normoxic and hypoxic H9C2 cells by NCBP3 IP. (B) The interaction between METTL3 and eIF4A2 in normoxic and hypoxic H9C2 cells with NCBP3 knockdown by reverse IP. (C‐F) The integrity of NCBP3, METTL3 and eIF4A2 in normoxic and hypoxic H9C2 cells with NCBP3 knockdown by gel filtration assay

In order to verify the reliability of mass spectrum, we pulled down NCBP3 and detected METTL3 and eIF4A2 by WB. First, we could determine that the interaction of NCBP3 with METTL3 and eIF4A2 was both substantially strengthened in hypoxic condition compared with normal control (Figure 2A). Next, we observed that NCBP3 deficiency indeed affected the interplay between METTL3 and eIF4A2 in hypoxic but failed in normoxic H9C2 cells (Figure 2B). Gel filtration assay indicated that METTL3 and eIF4A2 averagely dispersed everywhere in normoxic condition no matter whether NCBP3 was knockdown or not (Figure 2C,D), whereas METTL3, eIF4A2 and NCBP3 were concentrated within the same fraction in hypoxic H9C2 cells (Figure 2E) and dissipated after NCBP3 was knockdown (Figure 2F), indicating that METTL3, eIF4A2 and NCBP3 might constitute a hypoxia‐specific complex in cardiomyocytes.

### 3.3 The m6A profiling regulated by METTL3‐mediated RNA methylation in hypoxic H9C2 cells

**FIGURE 3:**
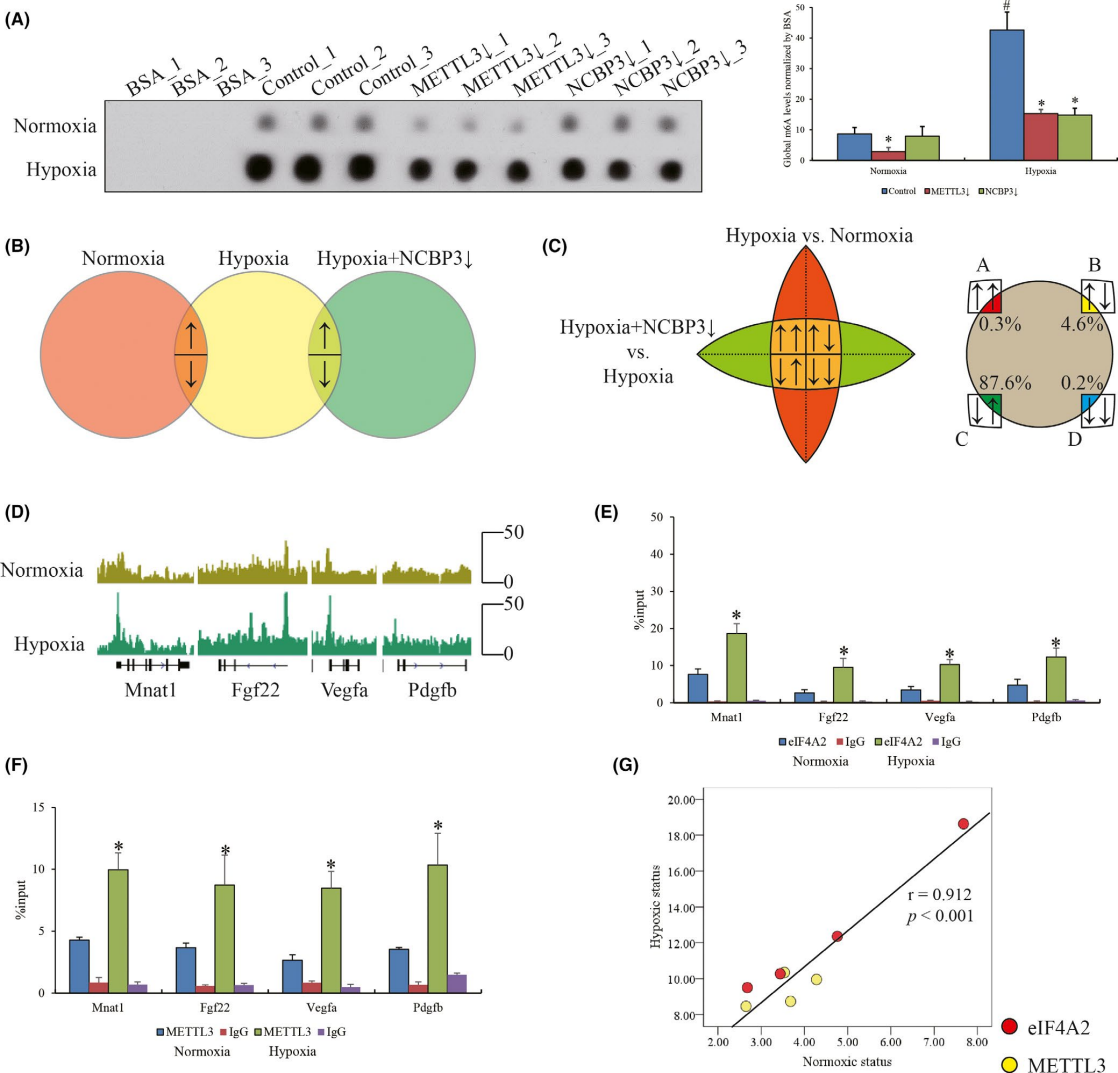
M6A RNA methylation responding to hypoxia regulated by NCBP3. (A) The global m6A RNA methylation in normoxic and hypoxic H9C2 cells with NCBP3 or METTL3 knockdown by dot plot assay. (B) The differential expressed genes of m6A RNA methylation MeRIP‐seq assay among normoxic and hypoxic H9C2 cells with NCBP3 knockdown by Venn diagram view. (C) The intersection between MeRIP‐seq and RNA‐seq of our previous data. (D) Gene browser views of m6A peaks on Mnat1, Fgf22, Vegfa and Pdgfb by Integrative Genomics Viewer. The enrichments of (E) eIF4A2 and (F) METTL3 on Mnat1, Fgf22, Vegfa and Pdgfb by RIP‐qPCR assay. ‘*’ represents the statistical significance. (G) The correlation between the enrichments of eIF4A2 and METTL3 on these target genes in normoxic and hypoxic condition by Pearson correlation analysis

Although METTL3 has been widely acknowledged as an essential enzyme for m6A RNA methylation, how METTL3 works with eIF4A2 in the current system is obscure. To this end, we conducted dot plot and MeRIP‐seq to investigate the potential connection between transcriptome‐wide m6A RNA methylation and eIF4A2 in hypoxic condition. We observed that m6A RNA methylation levels could be induced under hypoxia compared to normal control, but compromised by additional NCBP3 or METTL3 knockdown (Figure 3A). However, in normoxic condition, only METTL3 but not NCBP3 knockdown indeed reduced the global m6A RNA methylation (Figure 3A). Moreover, MeRIP‐seq data indicated the four profiles of m6A differentially enriched genes (log2FC >1 or <−1, *p* < 0.05) that were higher or lower in hypoxia compared with control, and combined with lower or higher compared with hypoxia plus NCBP3 knockdown (Figure 3B). Surprisingly, we notice that the profile C containing 85 genes with highly enriched m6A methylation in hypoxia and reduced in NCBP3 knockdown was 87.6% identical with DEGs of translatome data between normoxic and hypoxic H9C2 cells in our previous study^10^ (Figure 3C). In profile C, genes with differential m6A RNA methylation at 5’ UTR were all included in RNA‐seq data (Figure 3D ^),^ indicating that METTL3‐mediated m6A methylation at 5' UTR might play a regulatory role in translational process in hypoxic H9C2 cells. Furthermore, eIF4A2 and METTL3 RIP‐qPCR at 5' UTRs on Mnat1, Fgf22, Vegfa and Pdgfb (Figure 3E,F) confirmed that the changes in eIF4A2 enrichment on target mRNAs basically positively correlated with METTL3 at 5’ UTRs (*r* = 0.912, *p* < 0.001; Figure 3G). Taken together, our data determined that METTL3 contributed to m6A RNA methylation at 5' UTR to facilitate eIF4A2 location on target genes in hypoxic cardiomyocytes.

### 3.4 NCBP3‐METTL3‐eIF4A2 as a myocyte‐specific regulatory axis to regulate gene expression responding to hypoxia

**FIGURE 4:**
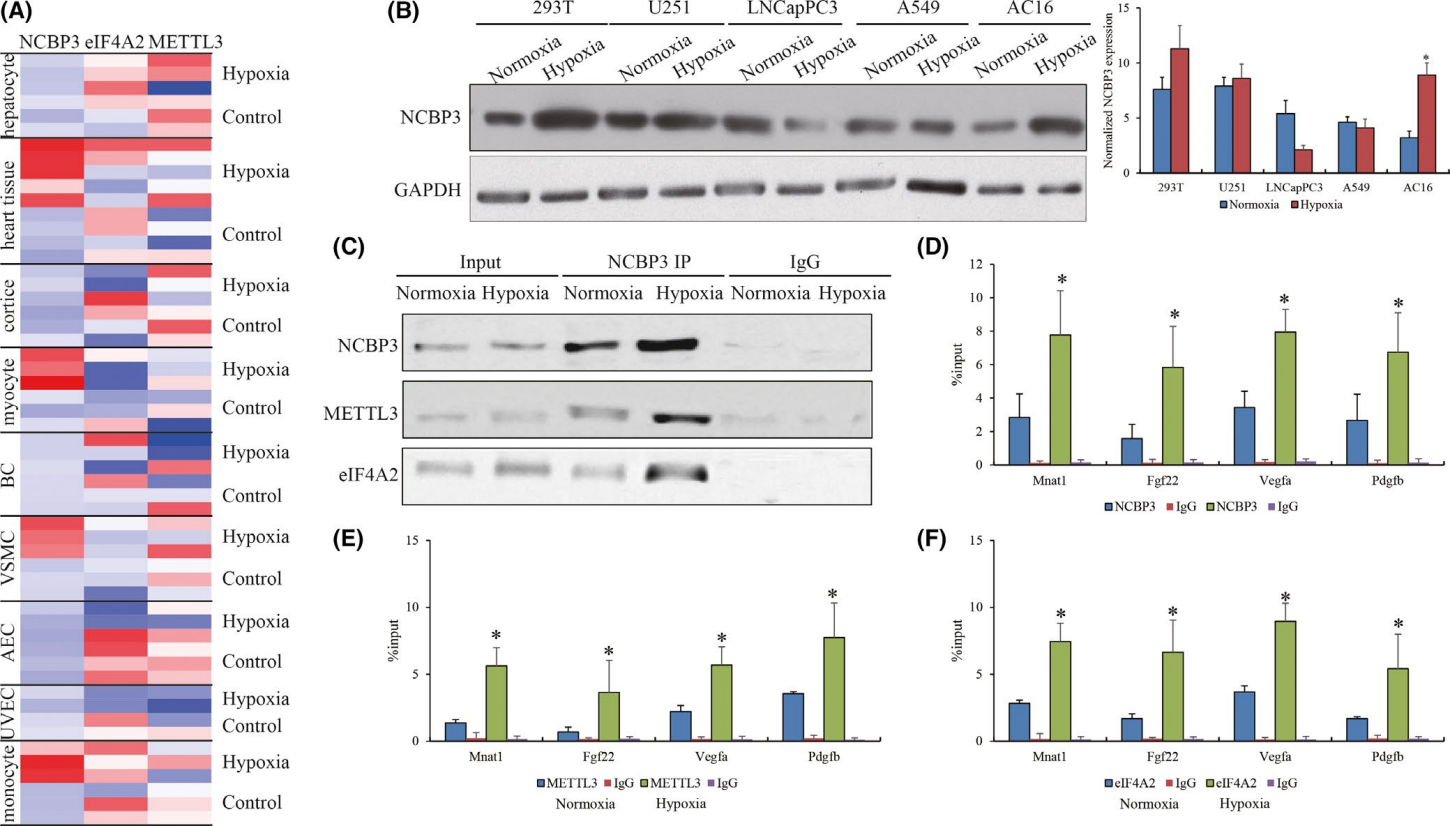
Cardiac‐specific axis of NCBP3/METTL3/eIF4A2 responding to hypoxia. (A) The heatmap view for the expression profiling of NCBP3, METTL3 and eIF4A2 in multiple tissues and cells undergoing hypoxic stress from the public RNA‐seq data. (B) NCBP3 expression in different types of cells with hypoxic stress. (C) Interaction of NCBP3 with METTL3 and eIF4A2 in normoxic and hypoxic human AC16 cells by NCBP3 IP. The enrichments of (D) NCBP3, (E) METTL3 and (F) eIF4A2 on Mnat1, Fgf22, Vegfa and Pdgfb by RIP‐qPCR assay. ‘*’ represents the statistical significance

Although the importance of NCBP3 for coordination with METTL3 and eIF4A2 in hypoxic H9C2 cells has been determined, whether this regulatory axis is conserved and can be extended to other human diseases associated with hypoxia is unknown. We accessed the high‐throughput sequencing on transcriptome of different tissue types undergoing hypoxic stress from public database. The normalized expressions of NCBP3, METTL3 and eIF4A2 were evaluated in human monocytes (GSE162834), human umbilical vein endothelial cells primary human aortic endothelial cells and smooth muscle cells (GSE154427), breast cancer (GSE167956), human cardiomyocytes (GSE144424), mouse brain cortices (GSE173544), mouse heart tissues (GSE169214) and mouse hepatocytes (GSE159320). We observed that NCBP3 was specially increased in monocytes, smooth muscle tissues and cardiomyocytes with hypoxic condition (Figure 4A), indicating that NCBP3 was likely to be a mesoderm‐derived tissue‐specific RNA binding protein. Consistently, we verified the expression of NCBP3 in human 293T embryonic kidney cells, glioma U251 cells, prostate cancer LNCapPC3 cells, non‐small‐cell lung cancer A549 cells and AC16 cardiomyocytes, and we found that NCBP3 was only highly expressed in AC16 with hypoxia treatment compared with normal control (Figure 4B). Consistent with H9C2 cell model, NCBP3 could also facilitate the recruitment of eIF4A2 and METTL3 in hypoxic AC16 cells (Figure 4C). Besides that, RIP‐qPCR was conducted to determine that the presence of robust occupancies of NCBP3, eIF4A2 and METTL3 at 5' UTR in human genes of Mnat1, Fgf22, Vegfa and Pdgfb, which were homologous with rat, also existed in human cardiomyocytes with hypoxic stress (Figure 4D).

Collectively, these results indicated that NCBP3 exerted as a special scaffold to drive the coordination between METTL3 and eIF4A2 in cardiomyocytes under the hypoxia stress.

## 4 DISCUSSION

There are multiple steps in the process of gene expression from eukaryotic nucleus to cytoplasm. The canonical cap‐binding complex comprised of NCBP1 and its binding partner NCBP2 is acquired co‐transcriptionally by the precursors of all mRNAs. As an alternative accessory protein for NCBP2, NCBP3 is also capable of binding m^7^G‐cap and recruiting NCBP1 via an RNase‐insensitive manner in vitro.^22^ Moreover, NCBP3 can also contribute to RNA splicing and exon–exon junctions coordinated with exon–junction complexes.^23^ In our mass spectrum data, the components of NCBP2, eIF4A3, MAGOH, RBM8A ALYREF and DDX39B are all identified both in normoxia and hypoxia H9C2 cells. But we further find the substantial interaction of eIF4A2 and METTL3 with NCBP3 especially in hypoxic stress, indicating a hypoxia‐specific role in cardiomyocytes although the potential connection of these novel binding proteins with NCBP3 is never studied. IP results confirm that the interaction among NCBP3, eIF4A2 and METTL3 is likely to only appear in a very particular system (hypoxic stress). In present study, we document that NCBP3 only exerts as a special scaffold to link with eIF4A2 and METTL3, but we have no idea about whether cap‐binding complex partakes in gene translation process under the hypoxic stress. Actually, there is a substantial interaction between METTL3 and eIF4A2 (with the same fraction in normoxic H9C2 cells by gel filtration), and NCBP3 can strengthen the connection of eIF4A2 and METTL3 in hypoxia. Here, we speculate that METTL3 and eIF4A2 may function individually although translational initiation factors or m6A RNA methylation may have some joint proteins. Previous studies have indicated the function of METTL3 can be affected by hypoxia/reoxygenation in myocytes^24^ and vascular smooth muscle cells,^25^ which implies that METTL3 is a downstream target of hypoxia for regulating gene expression. Unfortunately, all current researches including ours fail to figure out the mechanism behind it. Which key factors induced by hypoxic environment facilitate NCBP3 to enhance the affinity between and eIF4A2/METTL3 on earth needs to be addressed in future study.

N6‐methyladenosine (m6A) RNA methylation is prevalently and abundantly enriched in RNA in eukaryotic cells.^26^ A number of scholars pointed out that m6A RNA methylation has been found to be involved in multiple cellular processes, such as RNA maturation, alternative splicing and protein synthesis.^27^, ^28^ The growing evidence suggests that abnormally regulation of m6A may profoundly contribute to the carcinogenesis.^29^ A series of enzymes termed as m6A ‘writers’, ‘erasers’ and ‘readers’ based on the different functions have been documented to install m6A RNA methylation modification. The catalytic process of METTL3 has been considered the most common m6A pathway, especially in mRNA. The dysregulation of METTL3 has been determined to be implicated in many aspects of human diseases which have prompted many researchers to explore its possible molecular mechanism. METTL3 associated with ribosomes can promote translation via translation initiation machinery at 5' UTR^30^ or mRNA circularization at stop codon.^31^ In current case, we determine that METTL3 recruited by NCBP3 can improve m6A RNA methylation levels and then facilitate eIF4A2 for translation process. The other enzymes for m6A RNA methylation are not involved in post‐transcription regulatory complex modulated at 5' UTR by NCBP3. This is because the genes with differential translation compared with their transcription have a NCBP3 binding motif at 5' UTR, but it cannot exclude the putative functions of other enzymes such as YTHDF3 at exon–exon junctions or 3' UTR^32^ for regulating translation. We believe that the differential translation of a proportion of genes induced by hypoxia is supposed to respond to hypoxic stress by the synergic and complicate effects of multiple RNA‐binding proteins.

Overall, we unmasked NCBP3, a novel hypoxia‐specific response protein functions as a scaffold to coordinate METTL3 and eIF4A2 for enhancing gene translation by m6A RNA methylation in cardiomyocytes upon hypoxic stress. NCBP3 can be considered as a new therapeutic target for acute myocardial infarction prevention.

## CONFLICT OF INTERESTS

All the authors have declared no conflict of interest in this study.

## AUTHOR CONTRIBUTIONS

**Fei Ye:** Data curation (equal); Formal analysis (equal); Methodology (equal); Resources (equal). **Xiaoyan Wang:** Data curation (equal); Formal analysis (equal); Methodology (equal). **San Tu:** Data curation (equal); Formal analysis (equal); Investigation (equal); Methodology (equal). **Lixiong Zeng:** Data curation (equal); Formal analysis (equal); Software (equal); Validation (equal). **Xu Deng:** Data curation (equal); Formal analysis (equal); Methodology (equal); Software (equal). **Wenzhi Luo:** Data curation (equal); Formal analysis (equal); Investigation (equal); Methodology (equal); Software (equal). **Zhihui Zhang:** Conceptualization (lead); Funding acquisition (lead); Project administration (lead); Visualization (lead); Writing‐original draft (lead); Writing‐review & editing (lead).

## Supporting information

Figure S1

Table S1

Table S2

## Data Availability

The data that support the findings of this study are available from the corresponding author upon reasonable request.
