# Supplementary figures and images for: The effects of NCBP3 on METTL3‐mediated m6A RNA methylation to enhance translation process in hypoxic cardiomyocytes
Source: J Cell Mol Med. 2021 Aug 12;25(18):8920–8. doi: 10.1111/jcmm.16852 (PMC8435433; doi:10.1111/jcmm.16852)

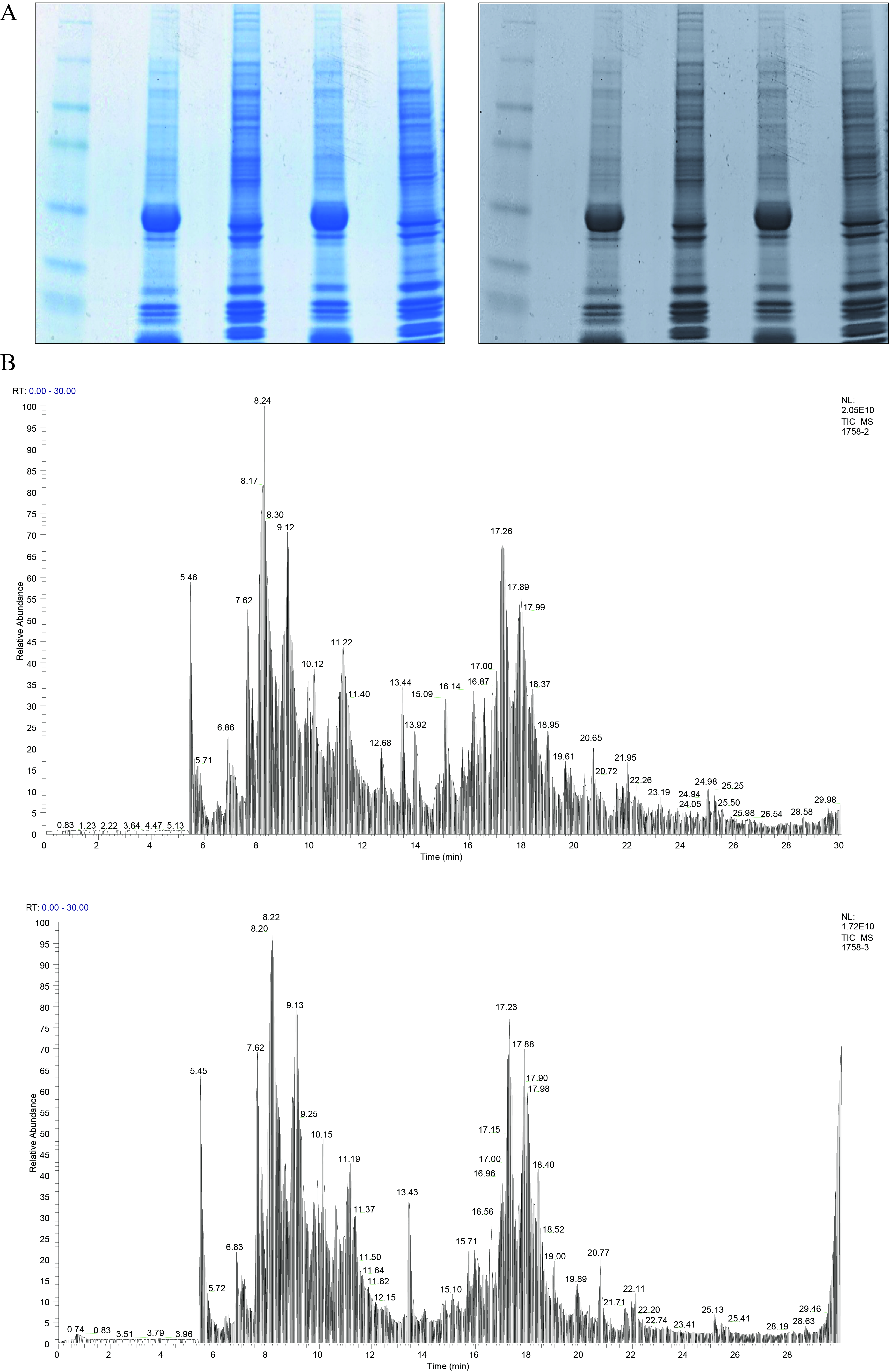

Supplement: Supplementary file 1 — Figure S1 [file JCMM-25-8920-s002.tif]
