# Supplementary material for: The effects of NCBP3 on METTL3‐mediated m6A RNA methylation to enhance translation process in hypoxic cardiomyocytes
Source: J Cell Mol Med. 2021 Aug 12;25(18):8920–8. doi: 10.1111/jcmm.16852 (PMC8435433; doi:10.1111/jcmm.16852)
Supplement: Supplementary file 2 — Table S1 [file JCMM-25-8920-s001.docx]

|  | Sequence |
| --- | --- |
| Mnat1 | 5’- AGGAACTGTAGCGGCTGGAACAGAGAACTC -3’ |
|  | 5’- ACAGTCCCAAGACTCACAGTGTGTGT -3’ |
| Fgf22 | 5’- GACAAACAGGAGCTGACAG -3’ |
|  | 5’- GATCCACACGCAGGAAAAAGTGGGT -3’ |
| Vegfa | 5’- AGGCCGCGGCGCTGGGGGCGAGCTGAG -3’ |
|  | 5’- CGCTCTCTGACCGGTCTCTCTCTCTCTC -3’ |
| Pdgfb | 5’- CGGCGCGCTCCGTCTACGCGTCC -3’ |
|  | 5’- GGCTCACGCGCTGCAAGGCTCCAAAGTTCA -3’ |

Table S1. All primers for RIP-seq used in this study are listed above.
